# Retroperitoneal Endometrioid Carcinoma Arising from Ureteral Endometriosis

**DOI:** 10.1155/2019/9273858

**Published:** 2019-06-10

**Authors:** Daiken Osaku, Fuminori Taniguchi, Maako Moriyama, Shinya Sato, Tetsuro Oishi, Tasuku Harada

**Affiliations:** Department of Obstetrics and Gynecology, Tottori University Hospital, Japan

## Abstract

Primary ureteral endometriosis is considered to be an uncommon form with an incidence of less than 0.1% in endometriosis. We reported a case of retroperitoneal endometrioid carcinoma possibly arising from ureteral endometriosis. A 52-year-old woman complained left backache. A solid mass in left retroperitoneal cavity with hydronephrosis was found. Diagnostic laparotomy with bilateral salpingo-oophorectomy and biopsy of the mass were performed. Although the primary lesion was not defined in the surgery, the histopathological diagnosis of specimen was endometrioid carcinoma. In the interval debulking surgery after the chemotherapy, tumor adjacent the left iliac vessels was observed. We resected the mass together with 5 cm left ureter and performed ureterectomy and anastomosis. The patient was diagnosed as the malignant transformation of ureteral endometriosis. Adjuvant chemotherapy is now undergoing. In case of finding the cancer tissue in women, we should take into account the malignant transformation of less common endometriosis, including ureteral endometriosis.

## 1. Introduction

Endometriosis is a common disease, which is characterized by the presence of endometrium-like tissues outside the uterus. It affects approximately 10% of women of reproductive age [1]. Endometriosis commonly forms lesions, such as peritoneal, ovarian, and deep infiltrating endometriosis, and less common site endometriosis consists of bowel, urinary tract, and distant sites, such as lung or umbilicus. Because of their rarity, the incidence, natural history and treatment efficacy of endometriosis at less common sites/rare sites are not fully understood. One of the most serious clinical problems in the management of endometriosis is malignant transformation. The development of carcinoma which could arise from ureteral endometriosis is a rare occurrence. Here we reported a case of retroperitoneal endometrioid carcinoma possibly transformed from ureteral endometriosis.

## 2. Case Report

**Figure 1:**
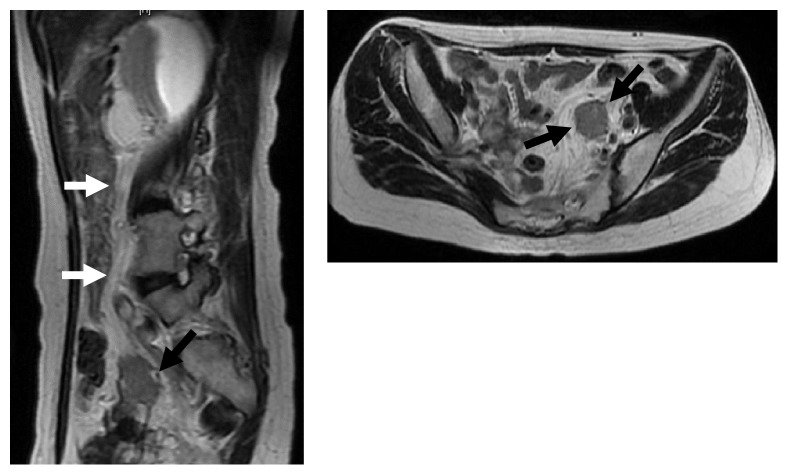
MRI findings of (a) the abdomen and (b) the pelvis with T2-weighted images: white arrows: left ureter and black arrows: a postperitoneal tumor.

A 52-year-old woman (gravida1 para1) without any past medical history visited Tottori Prefectural Central Hospital, and complained with left backache in January 17th, 2017, and she had several kinds of medical examinations by a urologist at that time. A CT scan showed a 4 cm mass in left retroperitoneal cavity and ipsilateral hydronephrosis above stenosis, however, the primary lesions could not be identified. Magnetic resonance imaging (MRI) of the abdomen and pelvic cavity also exhibited a 4 cm mass and left hydronephrosis (Figure 1). Several tumor markers, such as CA125 and CA19-9, were in normal ranges except for NCC-ST-439 (normal range: less than 4.5 U/mL). Diagnostic laparotomy with bilateral salpingo-oophorectomy, biopsy of left retroperitoneal tumor, and endometrial curettage were performed in April 13th, 2017. Because we predicted the severe adhesion in her pelvic cavity, the exploratory laparotomy was chosen instead of laparoscopic operation. A histological examination revealed the endometrioid carcinoma suspicious of an extrinsic origin. No malignant tissues were found in the ovary, fallopian tube and eutopic endometrium.

**Figure 2:**
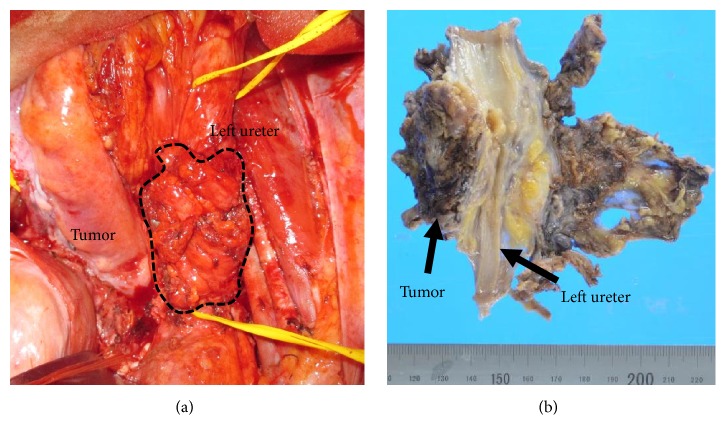
(a) The pelvic mass encasing with left ureter observed in surgery and (b) macroscopic the resected tissue after formalin-fixation.

**Figure 3:**
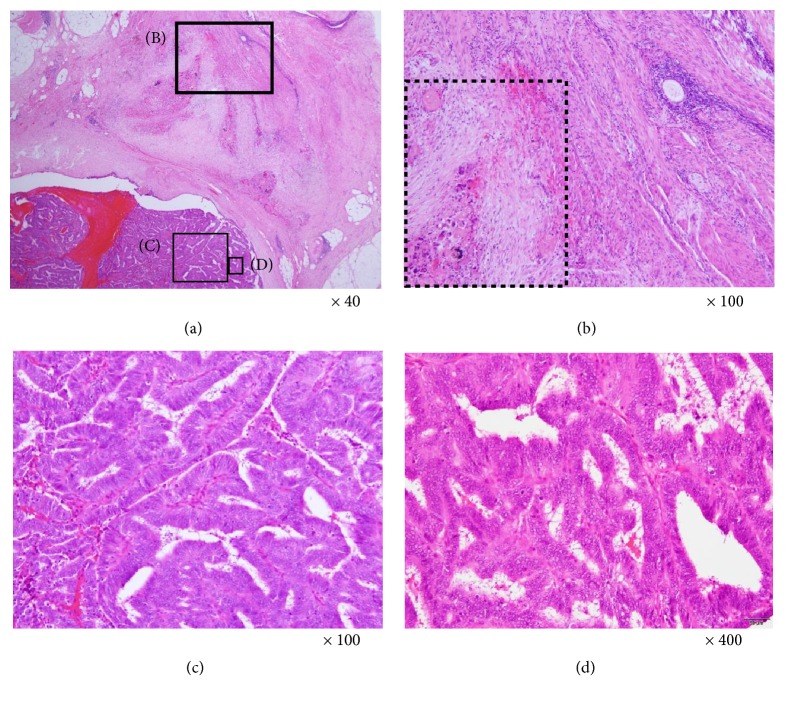
H.E. staining of the tissue diagnosed as the malignant transformation of ureteral endometriosis. (a) The endometrioid carcinoma adjacent to endometriotic tissue was observed (x40). In the photograph (a), the locations of (B) the endometriotic tissue with necrotic tissue found in the dotted area after the chemotherapy (x100), (C) the endometrioid carcinoma (x100), and (D) the atypical endometriosis (x400) were indicated.

She was referred and admitted to Tottori University Hospital as Cancer of unknown primary (CUP) in June 30th, 2017. The guidelines for treatment of uterine body neoplasm (the endometrioid type) by Japan Society of Gynecologic Oncology recommended the chemotherapy as adjuvant treatment for the case with the difficulty of excision. To prevent the metastasis in the other organs, we chose the systemic chemotherapy as a preoperative adjuvant treatment. First, 3 cycles of Paclitaxel and Carboplatin were administered, however, the size of tumor did not decrease. Thereafter, as the second-line regimen, she underwent 4 cycles of Doxorubicin and Cisplatin, and achieved approximately 28% decrease in the target lesion. Hence, we carried out the second surgery in February 1st, 2018, and found the tumor adjacent to left iliac vessels. Left ureter, sigmoid colon and uterus were involved with the tumor (Figure 2). Following adhesiolysis, we removed the tumor with approximately 5 cm ureter, and accomplished left ureterectomy, ureter anastomosis, hysterectomy, left pelvic lymphadenectomy, and omentectomy. However, we could not identify the primary malignant lesion. Pathological examination proved the tissue of ureteral endometriosis adjacent to the malignant tissues (Figures 3(a)–3(d)). The endometriotic tissue with necrotic tissue influenced possibly by the chemotherapy (Figure 3(b)), and the endometrioid adenocarcinoma (Figure 3(c)), and the part of atypical endometriosis, which are considered to be the precancerous lesion were observed (Figure 3(d)). These features were consistent with the diagnosis of well differentiated endometrioid adenocarcinoma (Grade 1 FIGO), surrounded with endometriotic tissue (Figure 3(a)). We diagnosed this patient as the malignant transformation of ureteral endometriosis. Posttreatment imaging showed no residual tumor. Since then, she was treated with 4 cycles of adjuvant chemotherapy with Doxorubicin and Cisplatin which had been effective, and the immediate postoperative condition was uneventful. The patient has not shown any findings of recurrence until this December 31st, 2018.

## 3. Discussion

Ureteral endometriosis (UE) occurs presumably in 0.1-1% of the cases of endometriosis [2]. It is mainly found incidentally during laparoscopy for extensive endometriosis [3]. The symptoms are usually non-specific, such as dysmenorrhea, dyspareunia and non-menstrual pelvic pain, and owing to secondary obstruction [4, 5]. The diagnosis of UE is difficult since the disease may be clinically silent in up to 30% of patients. Although the several radiological methods, such as MRI, Multi-slice computed tomography, and Intravenous pyelography, have been proposed, there is no consensus on which diagnostic technique should be used to assess UE. In general, MRI is superior to ultrasound or CT scan to evaluate the pelvic mass. With regard to the malignant transformation of ovarian endometrioma, a morphologic MR features suggestive malignancy is the solid enhancing mural nodule. In this case, it was difficult to determine the real tumor or the enlarged lymph nodes existed. UE most commonly affects a single distal segment of the ureter, with a left predisposition in most of the patients [3]. Patients may temporal benefit by medical therapy, however, surgery, such as conservative ureterolysis, radical ureterectomy or uretero-neocystostomy in relation to the type, site and length of ureteral involvement would be needed [2].

As far as we know, only two case reports have previously stated the malignant tumors that may have arisen from UE [6]. The first case showed a 48-year old patient presenting a retroperitoneal tumor and hydronephrosis with disseminated endometriosis, after 5-years supra-cervical hysterectomy and bilateral salpingo-oophorectomy for ovarian endometriotic cyst. At the surgery, the right ureter involved severely by a tumor was found. Pathologic examination verified adenosquamous endometrioid carcinoma [7]. In the second case, a 54-year old patient, after undergoing the same surgical treatment due to bilateral ovarian cysts and uterine fibroids, received unopposed hormonal treatment. This patient was not consistent with a gonadal origin. After several years, the patient presented a fixed mass encasing the right ureter. The histologic findings proved the ureteral infiltration of well-differentiated endometrioid adenocarcinoma [8]. In both cases, it was impossible to exactly define the origin of tumor, as both ureter and retroperitoneal structures were involved. In addition, as the previous two cases were treated with estrogen replacement therapy after surgery, further investigation regarding the association between unopposed hormonal stimulation and malignant transformation of extra-ovarian endometriosis would be needed.

Because of its rarity, the incidence, etiology and clinical management of the less common/rare site endometriosis-associated cancer is poorly understood. Although there are sporadic case reports about this type of cancer, little is known about the pathophysiology of the disease. In addition, there were no definite criteria to define malignant transformation from less common/rare site endometriosis. The ovary accounts for approximately 80% of malignancies associated with endometriosis, although the remaining cases are likely to occur in extragonadal sites. In terms of the malignant transformation of the ovary occurred in Japan, it was reported 33 cases of endometriosis-associated ovarian cancer, in which 23 (70%) cases were clear cell carcinoma and 8 (24%) were endometrioid carcinoma [9].

In up to 90% of patients of UE, major pathological types could be distinguished: extrinsic and intrinsic, occurring with a 1:4 ratio. Pathological types of UE in this patient were the extrinsic type, because the endometriotic tissues were not found inside ureter and eroded from outside ureter. In the criteria of Sampson & Scott [10], there are (i) presence of both benign and neoplastic endometrial tissues in the tumor, (ii) endometrial histology, (iii) absence of additional tumor, and (iv) the morphologic demonstration of benign endometriosis contiguous with the malignant tissue is a prerequisite for adjudication of a malignancy originating in endometriosis. In this case, 3 of 4 criteria were met. In terms of (iv) criterion, the tissue of atypical endometriosis and endometrioid carcinoma may be disappeared after the chemotherapy. In this case, the endometriotic tissue which had been formed firmly between the uterus and the retroperitoneum infiltrated extrinsically the left ureter, thereafter changed to the malignant foci.

This is the third case reported previously in the literature of a patient with endometrioid carcinoma arising from periuretal endometriosis, and the second case with this condition whose endometriosis is not consistent with a gonadal origin. This case prompts interesting issue with regard to the pathophysiology and clinical presentation of extra-ovarian endometriosis. In case of pelvic mass of CUP, especially when clear cell or endometrioid carcinoma exists, we should consider the malignant transformation of less common endometriosis including ureteral endometriosis.
